# Pharmacogenetic variations and clinical implications of actionable CYP2D6/CYP2C19 variants in Central Indian patients with common mental disorders

**DOI:** 10.3389/fphar.2025.1697866

**Published:** 2025-11-25

**Authors:** Santenna Chenchula, Atal Shubham, Rozatkar Abhijit, Tamonud Modak, Kohat Komal, Ratinder Jhaj, Singh Jitendra, Satyaprakash V., Sadasivam Balakrishnan

**Affiliations:** 1 Department of Pharmacology, AIIMS Bhopal, India; 2 Department of Psychiatry, AIIMS Bhopal, India; 3 Department of Translational Medicine, AIIMS Bhopal, India

**Keywords:** pharmacogenetics, CYP2D6, CYP2C19, antidepressants, common mental disorders, indian population, copy number variation, CPIC

## Abstract

**Introduction:**

Genetic polymorphisms in CYP2D6 and CYP2C19 significantly influence the metabolism, efficacy, and safety of antidepressant medications. Limited data exist on the prevalence of these actionable pharmacogenetic variants in the Central Indian population. This study aimed to determine the frequency of clinically actionable Tier-1 alleles, genotypes, and metabolizer phenotypes and to evaluate their clinical relevance in patients with common mental disorders (CMDs).

**Methods:**

A total of 509 adults diagnosed with depression and anxiety disorders and receiving SSRI & SNRI antidepressant therapy were enrolled from the Department of Psychiatry, AIIMS Bhopal. Genotyping was performed using the KASP qPCR assay, and CYP2D6 copy number variations (CNVs) were determined using the TaqMan qPCR assay.

**Results:**

Among the Central Indian cohort, the most frequent CYP2D6 alleles were *10 (21.6%), *41 (17.3%), and *4 (10.4%), while *3 (5.7%), *6 (1.9%). CNVs, Gene deletions(*5) and Gene duplications(xN) were detected in 4.2% and 4.1% of the cohort. For CYP2C19, the *2 (37.3%), *3 (2.3%), and *17 (16.1%) alleles were observed. Non-normal metabolizer phenotypes were present in 46.2% for CYP2D6 and 74.2% for CYP2C19; CYP2D6 ultra-rapid metabolizers accounted for 5.3%. Overall, 86% of participants had at least one clinically actionable pharmacogenetic phenotype. Overall, 7.5% of patients carried CYP2D6 variants and 20.6% carried CYP2C19 variants, for which CPIC guidelines recommend alternative drug selection or dose modification.

**Discussion:**

This study demonstrates a high prevalence of actionable CYP2D6 and CYP2C19 variants in the Central Indian population, underscoring the need for pharmacogenetic integration in psychiatric prescribing in Indian clinical settings, to enhance treatment efficacy and minimize adverse events.

## Introduction

Pharmacological management with antidepressants remains the cornerstone of treatment for common mental disorders (CMDs) such as major depressive disorder (MDD) and anxiety (Leichsenring et al., 2024). However, a significant proportion, around two-thirds, of patients do not attain remission with their initial antidepressant regimen (Nelson et al., 2008). High discontinuation rates are frequently attributed to inadequate therapeutic response or poor tolerability due to adverse effects, both of which contribute to suboptimal treatment outcomes (Nelson et al., 2008; Santenna et al., 2024). Genetic variability is a major contributor to interindividual differences in drug metabolism and treatment responses (Zanger and Schwab, 2013). Pharmacogenetic (PGx) testing has emerged as a valuable tool for guiding personalised medicine, in which treatment response and tolerability vary widely among individuals (Santenna et al., 2024; Bousman et al., 2023).

Polymorphisms in cytochrome P450 (CYP450) enzymes significantly influence an individual’s ability to metabolise and respond to many commonly prescribed medications (Zanger and Schwab, 2013). Among these, *CYP2D6* and *CYP2C19* are the two most important enzymes that bio-transform approximately 25%–35% of widely prescribed drugs, including many psychotropic medications, such as tricyclic antidepressants (TCAs), serotonin–norepinephrine reuptake inhibitors (SNRIs), selective serotonin reuptake inhibitors (SSRIs), and antipsychotics (Bousman et al., 2023; PharmGKB, 2024). Single-nucleotide polymorphisms (SNPs) and structural variations, such as copy number variations (CNVs), including gene deletions and duplications in *CYP2D6* and *CYP2C19,* can significantly alter enzyme function ranging from complete loss to increased activity and are responsible for a spectrum of metabolic phenotypes, including poor, intermediate, normal, rapid, and ultra-rapid metabolizers (Bousman et al., 2023; PharmGKB, 2024). With approximately 177- and 39-star (*) alleles, respectively, which have been catalogued by the Pharmacogene Variation Consortium (PharmVar; https://www.pharmvar.org/genes), both *CYP2D6* and *CYP2C19* are highly polymorphic enzymes (Bousman et al., 2023; PharmGKB, 2024). Many of these alleles are recognised as clinically actionable and have been designated as Tier 1 PGx variants by the Clinical Pharmacogenetics Implementation Consortium (CPIC) and through joint consensus guidelines by the Association for Molecular Pathology (AMP) Clinical Practice Committee’s Pharmacogenomics (PGx) Working Group (PharmGKB, 2024; Mastana, 2014).

The distribution of CYP2D6 and CYP2C19 polymorphisms varies substantially among global populations, primarily due to genetic diversity. India is home to over 4,500 anthropologically distinct groups and encompasses multiple ancestral clusters, including South, North, East, West, and Central Indian populations (Mastana, 2014). Central India, in particular, harbours a genetically unique set of 46 Scheduled Tribes (e.g., Gond, Bhil, and Baiga) that represent admixtures of Indo-European, Dravidian, and Austroasiatic ancestry patterns that are not typically observed in other Indian subgroups (Sharma et al., 2012). Despite this regional genetic distinctiveness, there is a critical lack of pharmacogenomics (PGx) data from Central India, which hinders the implementation of precision medicine in this area. While several studies have characterised *CYP2D6* and *CYP2C19* allele distributions in North and South Indian populations, data from Central India remain scarce. Even the IndiGenome initiative, a nationwide genome sequencing effort across India, represented this region with only 45 samples from the states of Madhya Pradesh and Chhattisgarh out of a total of 1,029, limiting population-specific PGx interpretation (Umamaheswaran et al., 2014; Jain et al., 2021).

To address this gap, the present study investigated the prevalence and clinical relevance of clinically actionable *CYP2D6* and *CYP2C19* alleles, genotypes, and predicted metabolizer phenotypes in individuals with common mental disorders from Central India. AMP Tier 1 *CYP2D6* alleles, such as *2, *3, *4, *5, *6, 10, 41, and Duplications and deletions (CNV) analysis and *CYP2C19* alleles, such as *2, *3, and *17, were the main targets of our genotyping approach (Zanger and Schwab, 2013; Bousman et al., 2023; PharmGKB, 2024). These variants collectively account for the most pharmacogenetically significant alterations that affect antidepressant metabolism and treatment outcomes. This study compared the study findings with data from other Indian subpopulations and worldwide cohorts, in addition to reporting allele and phenotypic frequencies. We also provide estimates of the proportion of individuals for whom deviations from standard prescribing practices are advisable, based on CPIC-guided metabolizer phenotype classifications. The findings support the integration of PGx-guided antidepressant prescribing into clinical practice and contribute to advancing precision medicine in the Indian healthcare setting.

## Methods

### Study participants

This cross-sectional study was conducted among patients attending the Department of Psychiatry, All India Institute of Medical Sciences (AIIMS), Bhopal, a tertiary care teaching institute, diagnosed with either MDD or anxiety by qualified psychiatrists using the Structured Clinical Interview for DSM-5 (SCID-5) and prescribed either SSRI or SNRI antidepressants. This standardised instrument ensured consistent and reliable diagnostic assessment across all study participants. Study subjects were adults aged 18–60 years of either sex and willing to give informed consent. All participants were informed about the study in their native language (Hindi), and written informed consent was obtained. The results of genotyping tests were revealed to participants upon their request. Reporting of study findings followed the STROBE (Strengthening the Reporting of Observational Studies in Epidemiology) guidelines for observational research (Cuschieri, 2019). The pharmacogenetic testing was conducted after clinical assessments and treatment initiation, ensuring that treating psychiatrists remained blinded to the genotyping results throughout the study period.

### Sample collection

After obtaining informed consent, a 3 mL blood sample was collected in ethylenediaminetetraacetic acid (EDTA) containing tubes from the study participants. Genomic DNA was extracted from the blood according to the manufacturer’s guidelines using the QIAamp^®^ DNA Blood Mini kit (Qiagen, Hilden, DE, USA) (Chacon-Cortes and Griffiths, 2014). Quantification and quality control were performed using a microplate spectrophotometer (Biotek Epoch2C, USA), and samples were stored at −20 °C until genotyping (Chacon-Cortes and Griffiths, 2014).

### Design of *CYP2D*6 and *CYP2C19* allele-specific KASP primers and SNP genotyping

Genotyping for clinically actionable tier one alleles, including *CYP2D6* *2 (rs16947), *3(rs35742686), *4 (rs3892097), *5, *6(rs5030655), 10 (rs1065852), and 41 (rs28371725), and *CYP2C19* alleles, such as *2 (rs4244285), *3 (rs4986893), and *17 (rs12248560), was done using the Kompetitive Allele-Specific PCR^®^(KASP^®^) SNP genotyping assay (LGC Biosearch Technologies, USA) on the QuantStudio™ 5 Real-Time PCR System (Applied Biosystems, USA). Data were analysed using QuantStudio Design and Analysis Software v2.8 and the Genotyping Analysis Module v1.5.2 (Applied Biosystems, USA) (Suo et al., 2020; He et al., 2014; Mayo et al., 2010).

Allele-specific primers were designed based on previously established principles and validated using the Primer-BLAST tool against the NCBI database (Ye et al., 2012). The validated sequences were submitted to LGC Biosearch Technologies for the standard KASP assay design using the proprietary Kraken™ software system (Suo et al., 2020). All assays were conducted in 96-well plates using a 10 µL reaction volume (5 µL KASP Master mix including 0.14 µL KASP assay Mix, 3 µL DNase- and RNase-free water, and 2 µL DNA containing ≥20 ng of gDNA) (He et al., 2014). We used a positive control sample with known genotypes and a No Template Control (NTC) in each run to monitor contamination.

### Copy number variation (CNV) analysis

*CYP2D6* CNV analysis was conducted using TaqMan CNV assays (Thermo Fisher Scientific, Waltham, MA, United States), targeting the exon 9 (Hs00010001_cn). The human RNase P assay (*Assay ID: 4403326, Life Technologies*), present in two copies per diploid genome, served as the internal reference gene to standardise DNA input and minimise assay-to-assay variability. A calibrator sample with a known diploid *CYP2D6* copy number (*n* = 2) was included to enable relative quantification using the comparative Ct (ΔΔCt) method. A No Template Control (NTC) was included in each run to monitor for contamination and non-specific amplification (Mayo et al., 2010). All reactions were run in replicates on the QuantStudio™ 5 Real-Time PCR System (Applied Biosystems) using 96-well plates with a final reaction volume of 10 μL, contained ≥20 ng of gDNA (2 µL), 5 µL of TaqMan^®^ Genotyping Master Mix, 0.5 µL of CNV assay mix, 0.5 µL of RNase P reference assay, and 2 µL of nuclease-free water, following the manufacturer’s protocol (Mayo et al., 2010). Data analysis was performed using CopyCaller™ software (Applied Biosystems, USA) using the comparative Ct (ΔΔCt) method (Schmittgen and Livak, 2008). In accordance with CopyCaller™ software, which assigns two quality metrics to each sample: a confidence value and an absolute Z-score, both are used as the primary criterion for accepting or rejecting copy number calls (Mayo et al., 2010; Schmittgen and Livak, 2008). Calls with Z-scores < ±1.75 were considered high confidence, those between ±1.75 and ±2.5 were interpreted with caution, and calls with Z-scores > ±2.5 were regarded as unreliable and either repeated or excluded (Mayo et al., 2010; Schmittgen and Livak, 2008). The confidence value (range: 0–1), with a value below 0.5 for the calibrator, even with correct copy numbers and an acceptable Z-score, will be logged as a potential model drift or assay inconsistency. Based on this analysis, CYP2D6 CNVs were classified as gene deletions (≤1 copy) or duplications (≥3 copies) (Wigle et al., 2023; Wang et al., 2024; Zeng et al., 2024). All CNV calls were subjected to stringent QC metrics, including Z-score and confidence thresholds as recommended by CopyCaller™ software. Low-confidence calls were either repeated or excluded from analysis, ensuring that only high-confidence results contributed to final phenotype assignments.

### CYP2D6 and CYP2C19 genotype identification and phenotype translation

Genotype calling and quality control were performed according to the manufacturer’s protocols. Genotypes were curated and validated using the standardised allele designation criteria and evidence levels provided by the P450 nomenclature maintained in the PharmVar database (https://www.pharmvar.org/genes), and cross-verified using ClinPGx’s Genotype Selection Interface (https://www.clinpgx.org/genotype) (Caudle et al., 2020; ClinPGx). Genotype-to-phenotype translation was performed using the CPIC guidelines (Bousman et al., 2023). For *CYP2D6*, phenotypes were assigned using the CPIC-recommended activity score (AS) system (https://www.clinpgx.org/page/cpicFuncPhen) (Caudle et al., 2020). In this system, each allele is assigned a numerical value based on its functional status, and the individual’s metabolizer phenotype is predicted using the overall activity score, which is determined by summing the values of the two alleles (diplotype) (Caudle et al., 2020). *CYP2D6* alleles are classified as follows: normal-function alleles (*1, *2, *2A) are assigned an AS of 1, while decreased-function alleles such as *10 and *41 are given AS values of 0.25 and 0.5, respectively, as per CPIC guidelines (Caudle et al., 2020). Furthermore, duplication of a *CYP2D6* functional allele (*1/*2, etc.) results in increased expression of the active enzyme. Based on the total activity score, the predicted metabolizer phenotypes are defined in the CPIC PGx as well as ClinPGx dosing guidelines as follows: ultrarapid metabolizers (UMs; AS > 2.25), typically having multiple copies of normal function alleles (e.g., CYP2D6*1/*1xN, *1/*2xN, *2/*2xN); normal metabolizers (NMs; AS = 1.25–2.25), with two normal function alleles (e.g., CYP2D6*1/*1, *1/*2, *2/*2); intermediate metabolizers (IMs; AS = 0.25–1.0), with one non-functional and one normal function allele or two decreased function alleles (e.g., CYP2D6*1/*5, *1/*4, *1/*3, *4/*10, *4/*41, *10/*10, *10/*41); and poor metabolizers (PMs; AS = 0), who carry two non-functional alleles (e.g., CYP2D6*3/*4, *4/*4, *5/*5, *5/*6). Predicted phenotypes were derived from diplotype data using manual curation guided by CPIC AS systems and functional allele classifications, and further verified by ClinPGx’s Genotype Selection Interface (GSI) (Bousman et al., 2023; Caudle et al., 2020; ClinPGx).

In contrast, there is no activity score system for *CYP2C19*; phenotype prediction is based on the functional classification of individual alleles (https://www.clinpgx.org/page/cyp2c19RefMaterials). The CYP2C19*1 is a normal function allele, *2 and *3 are non-function alleles, and *17 is an increased function allele linked to an enhanced enzymatic activity. The following are the corresponding phenotypes, including normal metabolizers (NMs; two normal function alleles: CYP2C19*1/*1), poor metabolizers (PMs; two non-functional alleles; CYP2C19*2/*2, *2/*3, *3/*3), intermediate metabolizers (IMs; one non-functional and one normal or increased function allele; CYP2C19*1/*2,*17/*2, *3/*17), rapid metabolizers (RMs: one normal-functional and increased function allele; CYP2C19*1/*17), and ultrarapid metabolizers (UMs; two increased function alleles: CYP2C19*17/*17). For *CYP2C19*, phenotypes were assigned directly from allele combinations as per CPIC functional classifications and further verified by ClinPGx’s Genotype Selection Interface (GSI) (Bousman et al., 2023; Caudle et al., 2020; ClinPGx).

### Identification of actionable PGx findings

Participants were classified as having a clinically actionable PGx phenotype if they exhibited a non-normal metabolizer phenotype IM, PM, RM, or UM for which CPIC prescribing guidelines recommend deviations from standard dosing, such as dose reduction, escalation, or drug substitution.

### Sample size

The sample size was determined based on the reported prevalence of *CYP2D6* alleles in previously published Indian studies. Among these, the lowest reported frequency was for CYP2D6*10 (5.2%), while the highest was for CYP2D6*2 (56%). The minimum required sample size was calculated using OpenEpi (version 3.01) for a single proportion. Assuming an anticipated prevalence of 5%, an absolute precision of 2% at a 95% confidence level (Z = 1.96), the estimated sample size was 474 participants (Sullivan et al., 2009). To enhance statistical power and ensure adequate representation of allele frequency variation, a total of 509 participants were recruited for this cross-sectional analysis.

### Statistical analysis

Descriptive and inferential statistical analyses were performed to evaluate the prevalence of *CYP2D6* and *CYP2C19* alleles, genotypes, and predicted metabolizer phenotypes. Allele frequencies were calculated using standard population genetics formulas, and results were expressed as frequencies and percentages. Genotype distributions were assessed for compliance with Hardy–Weinberg equilibrium (HWE) using both the chi-square (χ^2^) goodness-of-fit test and the exact test, as recommended by Wigginton et al. (2005) (Wigginton et al., 2005). To account for multiple testing, the Bonferroni correction was applied based on the number of variants tested per gene (α = 0.05/n). For CYP2D6, with eight variants tested, the corrected significance threshold was α = 0.00625, and for CYP2C19, with three variants tested, the threshold was α = 0.0167. After Bonferroni correction, p-values below the respective thresholds were considered statistically significant, indicating deviation from HWE, while p-values above the thresholds indicated conformity with HWE expectations.

Phenotype distributions are presented as descriptive prevalence estimates within the study cohort; as no statistical comparisons between groups were performed for phenotype frequencies, multiple testing correction was not applicable for these descriptive results. Metabolizer phenotypes, including clinically actionable deviations from standard dosing such as dose reduction, escalation, or drug substitution, were reported as proportions.

The proportion of individuals carrying ≥1 actionable phenotype across CYP2D6 and CYP2C19 was estimated as one minus the probability of having a wild-type genotype across both genes analysed, representing the joint probability of being a normal metabolizer at both loci. This approach assumes independence between CYP2D6 (chr22) and CYP2C19 (chr10) and the absence of linkage disequilibrium between them in Indian populations, consistent with prior pharmacogenomic prevalence studies (Zanger and Schwab, 2013; Bousman et al., 2023; Umamaheswaran et al., 2014; Chanfreau-Coffinier et al., 2019). All statistical analyses and data visualisations were performed using Microsoft Excel and R statistical software (version 4.5.1).

## Ethics statement

Ethical approval for this study was obtained from the Institutional Human Ethics Committee (Student Research), AIIMS, Bhopal, India *(Approval No. IHEC-SR/PhD/July/22)*. The study was conducted following the ethical principles outlined in the Declaration of Helsinki, the Indian Council of Medical Research (ICMR) National Ethical Guidelines for Biomedical and Health Research Involving Human Participants, 2017, the International Conference on Harmonisation Good Clinical Practice (ICH-GCP) guidelines, and the Organisation for Economic Co-operation and Development (OECD) Good Laboratory Practice (GLP) standards.

## Results

### Demographic and clinical characteristics of the study population

A total of 509 participants with CMDs were enrolled in the study from various districts of the Central Indian states of Madhya Pradesh and Chhattisgarh, reflecting broad regional coverage (Supplementary Figure S1). Among them, 370 (72.7%) were diagnosed with anxiety disorders and 139 (27.3%) with major depressive disorder (MDD). The mean age was 33.8 ± 11.1 years, with a median age of 32 years (interquartile range [IQR]: 25–41 years). Among the participants, 57.9% were male (n = 295) and 42.1% were female (n = 214). The average body mass index (BMI) among participants was 25.24 ± 2.96 kg/m^2^. The majority of participants were from urban areas (76.4%, n = 384), with a smaller proportion residing in rural regions (23.6%, n = 120). The most commonly prescribed antidepressants were sertraline (34.1%) and escitalopram (28.2%), followed by paroxetine (21.2%), venlafaxine (8.7%), and fluoxetine (7.8%) (Table 1).

**TABLE 1:** Demographic and clinical characteristics of the study population (N = 509).

Characteristic	Value
Age, years (mean ± SD)	33.8 ± 11.1
Age, years (median, IQR)	32 (25–41)
Sex, n (%)	
Male	295 (57.9%)
Female	214 (42.1%)
BMI, kg/m^2^ (mean ± SD)	25.24 ± 2.96
Residence, n (%)	
Urban	384 (76.4%)
Rural	120 (23.6%)
Marital status, married, n (%)	302 (59.3%)
Education, years (mean ± SD)	12.9 ± 4.1
Education, years (median, IQR)	15 (12–17)
Employment status, n (%)	
Employed	175 (34.4%)
Housewives	144 (28.3%)
Students	125 (24.5%)
Business	43 (8.5%)
Farmers	22 (4.3%)
CMD Diagnosis, n (%)	
Anxiety disorders	370 (72.7%)
Major depressive disorder	139 (27.3%)
Family history of psychiatric illness, n (%)	82 (16.1%)
Non-psychiatric comorbidity, n (%)	74 (14.5%)
Hypertension	31 (6.1%)
Hypothyroidism	25 (4.9%)
Type 2 diabetes mellitus	18 (3.5%)
Substance use, n (%)	105 (20.6%)
Alcohol	37 (7.2%)
Nicotine chewing	25 (4.9%)
Smoking	24 (4.7%)
Most prescribed SSRI/SNRI antidepressants, n (%)	
Sertraline (CYP2C19)	174 (34.1%)
Escitalopram (CYP2C19)	144 (28.2%)
Paroxetine (CYP2D6)	108 (21.2%)
Venlafaxine (CYP2D6)	44 (8.7%)
Fluoxetine (CYP2D6)	40 (7.8%)

CMDs, Common Mental Disorders; MDD, major depressive disorder; BMI, body mass index; IQR, Interquartile Range.

SD, standard deviation.

### Prevalence of clinically actionable CYP alleles and CNVs

**FIGURE 1:**
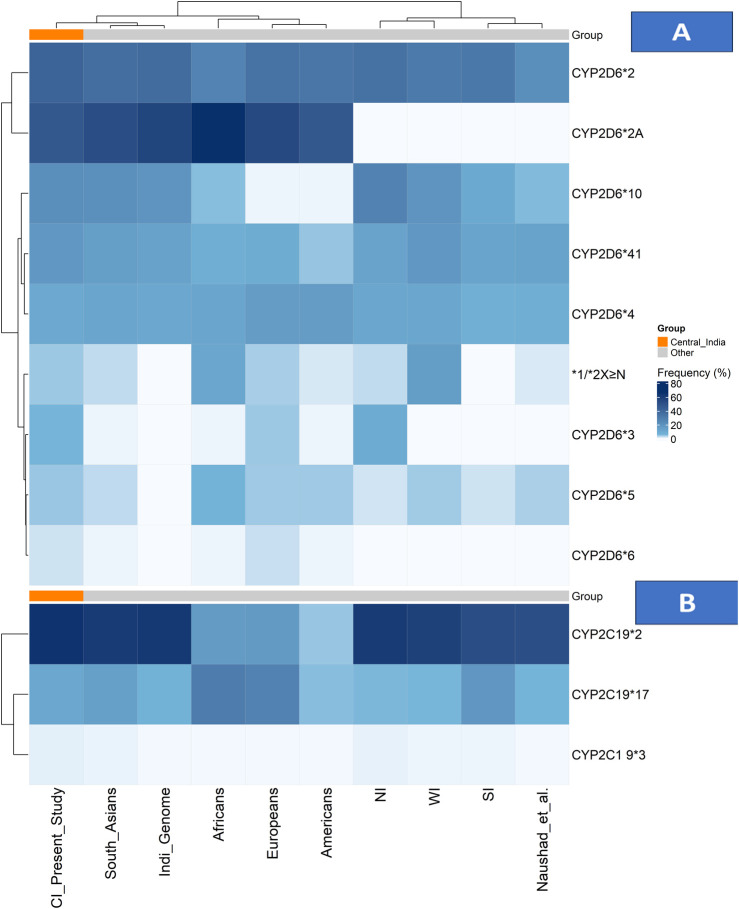
Allele frequency distributions of CYP2D6 and CYP2C19 across populations. **(A)** Heatmap showing CYP2D6 allele frequencies and **(B)** heatmap showing CYP2C19 allele frequencies across the Central Indian cohort, other Indian subpopulations, and global reference populations. Colour intensity represents allele frequency (white = low, dark blue = high), with hierarchical clustering applied to both alleles and populations. The Central Indian cohort is highlighted by an orange annotation bar in both panels. Abbreviations: CI: Central Indian (current study); NI: North Indian; SI: South Indian; WI: Western Indian.

In our study population, the minor allele frequencies (MAFs) of clinically actionable CYP2D6 and CYP2C19 variants revealed that the normal-function alleles CYP2D6*2 and *2A were observed at frequencies of 40.9% and 46.8%, respectively. Among the decreased-function alleles, CYP2D6*10 was prevalent in 21.6% of individuals, while CYP2D6*41 was found in 17.3% of the cohort. The non-functional alleles had a lesser prevalence: CYP2D6*4 in 10.4%, CYP2D6*3 in 5.7%, and *CYP2D*6*6 in 1.9% of the participants, respectively. The distribution of genotypes across CYP2D6 alleles varied in terms of wild-type (wt/wt), heterozygous (wt/mt), and homozygous mutant (mt/mt) forms. CYP2D6*2A had the largest percentage of homozygous mutant genotypes (29.7%), followed by CYP2D6*2 (16.1%) and CYP2D6*10 (10.8%) (Figure 1; Supplementary Table S1).

CNVs in the *CYP2D6* gene were observed in 17.4% (n = 89) in the cohort, comprising both gene deletions (CYP2D6*5) and duplications with amplified alleles including *CYP2D6* *1, *2, *4,* and *10. The frequency of the CYP2D6*5 allele was 4.2%, and the allele frequency of *CYP2D6* gene multiplications (*xN*) was 4.1% of the study cohort. The distribution of duplicated CYP2D6 alleles in the cohort included CYP2D6*2xN in 5.3% of individuals, *1xN in 0.6%, *4xN in 1.7%, and *10xN in 1.9%. Among these, 4.9% of participants harboured three gene copies, and 5.7% had four or more copies (Figure 1; Supplementary Table S2). All CNV calls passed stringent QC criteria, with only high-confidence calls included in the final analysis.

The frequency of the decreased-function CYP2C19*2 allele was 37.3%, but the frequency of CYP2C19*3 was lower at 2.3%. The increased function CYP2C19*17 allele was identified in 16.1% of individuals within the study population. The prevalence of genotypes of *CYP2C19* are as follows: wild-type genotypes (wt/wt) of CYP2C19*2 were seen in 44.8%, *3 in 97.6%, and *17 in 72.0%, respectively, while Heterozygous genotypes (wt/mt) were found in 35.8% (*2), 3.1% (*3), and 23.9% (*17), while homozygous mutants (mt/mt) seen in 19.4% (*2), 0.8% (*3), and 4.1% (*17). (Figure 1;Supplementary Table S3).

Genotype distributions for the majority of alleles were confirmed to the Hardy-Weinberg equilibrium, except for CYP2D6*2A, *10, *CYP2C19* *2 and *3, which showed significant deviation in HWE, characterised by a notable deficit of heterozygotes. The consistent pattern of heterozygote deficit across these alleles is a hallmark of the Wahlund effect, providing strong statistical evidence for substantial population substructure within this Central Indian cohort, likely due to the genetic distinctiveness and historical endogamy of its constituent tribal and caste groups (Sharma et al., 2012; De Meeûs, 2018).

### Clinically actionable diplotypes (genotypes) and metabolizer phenotype frequencies

Among the study population, 53.8% were classified as normal metabolizers (NMs) for *CYP2D6,* carrying either two normal-function alleles or one normal and one decreased-function allele. Among these individuals, the most frequently observed genotypes were CYP2D6*2/*41 (20.03%) and *2/*10 (17.7%), followed by *1/*2 (4.5%), *1/*10 (3.1%), *1/*41 (2.9%), *2/*2 (1.6%), 1/*1 (1.4%), *10x≥3/*1 (1.6%), and other genotypes were present each at ≤1 % only. Intermediate metabolizers (IMs) of *CYP2D6* comprised 38.7% of the study cohort. These individuals carried one decreased-function or one non-functional allele along with a functional allele, leading to reduced enzyme activity. Common genotypes in this group included CYP2D6*2/*4 (11.5%), *2/*3 (7.4%), *2/*5 (6.6%), *4/*10 (2.3%), *2/*6 (2.3%), and *3/*41 (1.6%). Poor metabolizers (PMs) of *CYP2D6*, defined by the presence of two non-functional alleles, represented 2.2% of the population. The observed genotypes were CYP2D6*4/*4 (0.6%), *5/*5 (0.6%), *3/*4 (0.4%), *3/*6 (0.4%), and *3/*5 (0.2%). Ultrarapid metabolizers (UMs) of *CYP2D6*, characterised by multiple copies of functional alleles, made up 5.3% of the participants. The most frequently observed duplicated genotypes were CYP2D6*1/*2X ≥ 3 and *2/*2X ≥ 3, each found in 1.7% of participants, followed by *41/*2X ≥ 3 at 1.5% (Table 2; Supplementary Table S4).

**TABLE 2:** Distribution of CYP2D6 and CYP2C19 Diplotypes and Phenotypes in the study cohort (n = 509).

Predicted phenotype	Genotypes	n (%)
CYP2D6		
Normal Metabolizer (NM)	*2/*41, *2/*10, *1/*2, *1/*10, *1/*41,*1/*1, *2/*2, *etc.*	274 (53.8%)
Intermediate Metabolizer (IM)	*2/*3, *2/*4, *2/*5, *2/*6, *4/*10, *3/*41, *10/*41, *etc.*	197 (38.7%)
Poor Metabolizer (PM)	*4/*4, *5/*5, *3/*4, *3/*5, *3/*6	11 (2.2%)
Ultrarapid (UM)	*1/*2X ≥ 3, *2/*2X ≥ 3, *41/*2X ≥ 3, *1/*1X ≥ 3	27 (5.3%)
CYP2C19		
Normal Metabolizer (NM)	*1/*1	131 (25.7%)
Intermediate Metabolizer (IM)	*1/*2, *2/*17, *1/*3, *3/*17	208 (40.9%)
Poor Metabolizer (PM)	*2/*2, *3/*3, *3/*3	94 (18.4%)
Rapid Metabolizer (RM)	*1/*17	65 (12.8%)
Ultrarapid Metabolizer (UM)	*17/*17	11 (2.2%)

In the case of *CYP2C19,* 25.7% of individuals were categorised as normal metabolizers (NMs), carrying two normal-function alleles, predominantly the CYP2C19*1/*1 genotype, whereas 40.9% of the participants were intermediate metabolizers (IMs), who usually carried one non-functional allele along with a normal or increased-function variant. The most common genotypes in the IM category were CYP2C19*1/*2 (25.7%), *2/*17 (11.6%), *1/*3 (2.8%), and *3/*17 in <1% of the cohort. Poor metabolizers (PM) of *CYP2C19* were observed in 18.4% of the study cohort. The most frequent genotypes were CYP2C19*2/*2 (17.4%), followed by *2/*3 and *3/*3, both seen in less than 1% of participants. Rapid metabolizers (RM) of *CYP2C19* constituted 12.8% of the study population and were defined by the CYP2C19*1/*17 genotype, while 2.2% were ultrarapid metabolizers (UMs), carrying the *17/*17 genotype, indicating increased enzyme activity. Collectively, among the study population, a total of 46.2% of participants carried non-normal *CYP2D6* metabolizer phenotypes (i.e., IM, PM, or UM), and 74.2% had non-normal *CYP2C19* phenotypes, emphasising the potential utility of PGx-guided prescribing. (Table 2; Supplementary Table S4).

The combined prevalence of individuals carrying at least one actionable pharmacogenetic genotype or predicted non-normal metabolizer phenotype for either *CYP2D6* or *CYP2C19* was estimated to be 86.2%.

## Discussion

This study presents the first comprehensive analysis of the prevalence and clinical relevance of clinically actionable *CYP2D6* and *CYP2C19* alleles, genotypes, and predicted metabolizer phenotypes in a Central Indian population of 509 individuals diagnosed with common mental disorders. Our findings contribute to the growing body of evidence indicating a high prevalence of pharmacogenetically actionable genotypes across globally diverse populations.

### Generalizability of allele and phenotype frequencies from the study population

Evidence from numerous studies has linked the pathophysiology of CMDs to polygenic influences involving genes such as *5-HTT*, *MAOA*, *APOE*, and *COMT* (Wu et al., 2019; Alshaya, 2022). In contrast, *CYP2D6* and *CYP2C19* are pharmacokinetic genes that primarily influence drug metabolism rather than disease susceptibility. Current evidence from large genome-wide association studies (GWAS) does not support a causal or enrichment association between *CYP2D6* or *CYP2C19* variants and psychiatric disorders such as major depressive disorder (MDD) or anxiety disorders (Taylor et al., 2020; Sun et al., 2025; Flint, 2023; Friligkou et al., 2024). Major pharmacogenomic guidelines and meta-analyses consistently apply *CYP2D6* and *CYP2C19* phenotype classifications across diverse disease populations, indicating that the observed allele distributions are reflective of background genetic variation rather than disease-specific selection (Milosavljević et al., 2021; Baldacci et al., 2023). Furthermore, our exploratory principal component analysis (PCA) based on CYP2D6 and CYP2C19 allele frequency data demonstrated clustering of our cohort within the broader South Asian genetic landscape, with a slight offset consistent with regional heterogeneity (Supplementary Figure S2). Therefore, the frequencies identified in our CMD cohort likely represent the broader population in this region and support the relevance of implementing pharmacogenomic-guided prescribing strategies in Central India.

### CYP2D6 allele distribution and metabolizer phenotypes comparison with other global populations

The *CYP2D6* gene, although contributing only 2% of the total hepatic enzyme volume, is responsible for the metabolism of nearly a quarter of commonly used medicines in clinical practice, including antidepressants, antipsychotics, opioid analgesics, and beta blockers (Bousman et al., 2023). Our combined heatmap analysis (Figure 1) reveals distinct patterns in the *CYP2D6* genetic architecture of Central India compared with global and regional populations.

Among the *CYP2D6* star alleles, the normal function alleles CYP2D6*2 and *2A were predominant (40.9% and 46.8%, respectively), with frequencies closely aligning with South Asian populations but showing distinct gradients compared with other continental groups (Kane, 2021; Gene-specific Information Tables for CYP2D6). Within India, a comparative analysis reveals notable regional variation, with CYP2D6*2 frequencies ranging from 21.4% to 37.8% across studies (Jain et al., 2021; Sivadas et al., 2024). In contrast, our Central Indian cohort exhibits distinct clustering patterns, as shown in Figure 1; Supplementary Table S1.

The nonfunctional alleles reveal clinically significant patterns, with CYP2D6*4 (10.4%) and *3 (5.7%) contributing substantially to the population burden. As shown in the heatmap, the *3 allele frequency in Central India (5.7%) exceeds that of most global populations (Kane, 2021; Gene-specific Information Tables for CYP2D6) and demonstrates a north–south gradient within India, being undetectable in South Indian studies but present at 9.2% in North Indians (Umamaheswaran et al., 2014; Paradkar et al., 2018). The CYP2D6*4 allele frequency (10.4%) is consistent with the IndiGenomes dataset (10.9%) (Jain et al., 2021) but higher than the 7.8% reported by Sivadas et al. (Sivadas et al., 2024), suggesting regional genetic substructure (Figure 1; Supplementary Table S1).

The decreased-function alleles CYP2D6*10 and *41 was particularly prevalent (20.9% and 17.3%, respectively). The *10 allele frequency aligns closely with South Asian estimates (21%) but is markedly higher than in other global populations (2%–6%) (Kane, 2021; Gene-specific Information Tables for CYP2D6). Regional analyses across India demonstrate substantial variability, with CYP2D6*10 frequencies ranging from 10.2% in South Indians to 27.2% in North Indians (Umamaheswaran et al., 2014; Wu et al., 2019), positioning Central India within an intermediate range, as shown in Figure 1; Supplementary Table S1.

A critical finding from our CNV analysis was the detection of gene duplications in 4.1% of participants—an area seldom addressed in Indian pharmacogenomics research. As shown in Figure 1; Supplementary Table S2, this prevalence places Central India between North Indian (2.5%) and Western Indian (15.4%) populations, reflecting the region’s unique genetic ancestry and historical migration patterns (Mastana, 2014; Sharma et al., 2012; Sivadas et al., 2024; Paradkar et al., 2018; Gaedigk et al., 2017).

**FIGURE 2:**
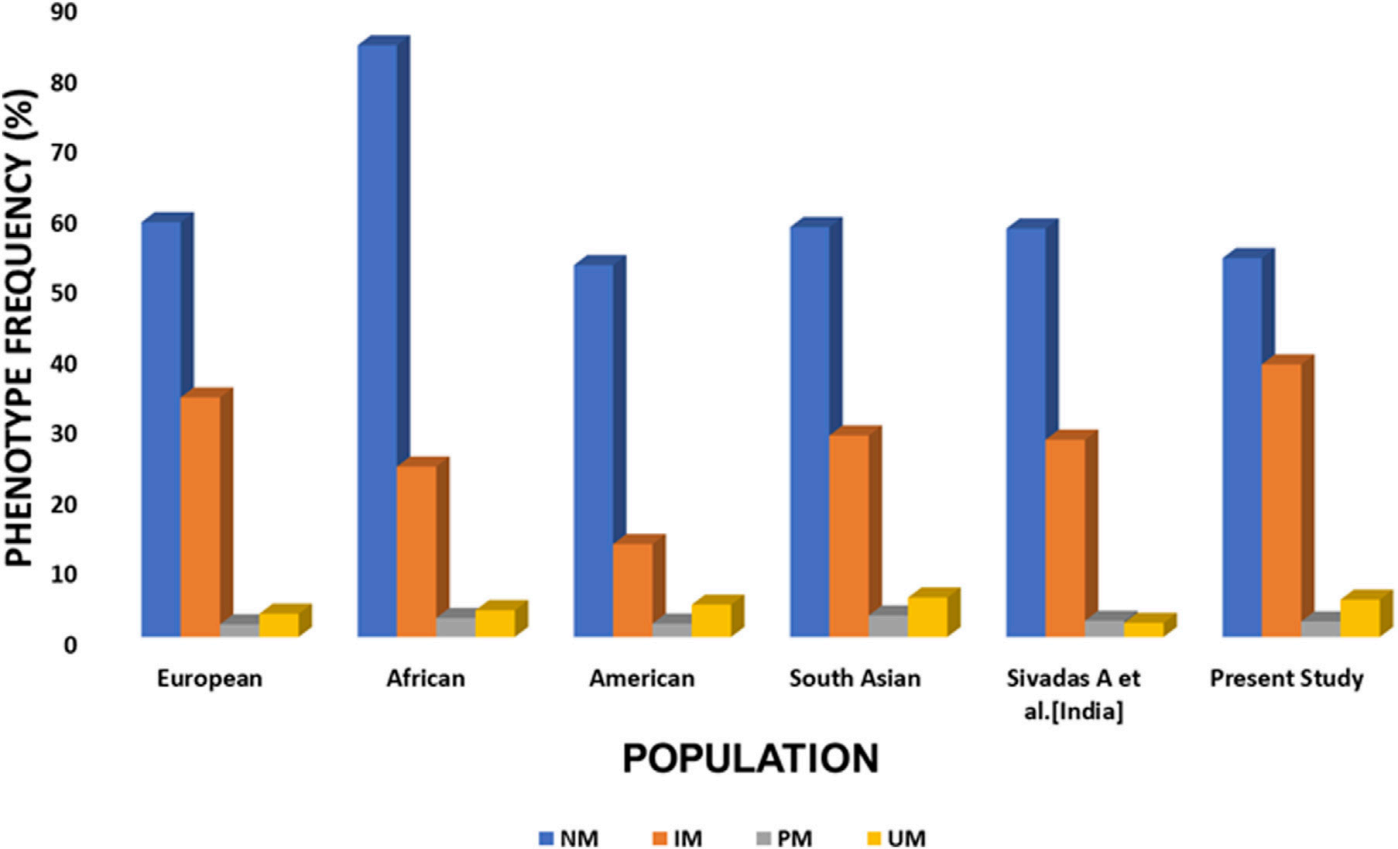
CYP2D6 phenotype distribution across populations. Grouped bar plot showing the proportion of normal metabolizer (NM), intermediate metabolizer (IM), poor metabolizer (PM), and ultrarapid metabolizer (UM) phenotypes for CYP2D6 across the Central Indian cohort, other Indian populations, and global populations. Abbreviations: CI: Central Indian (current study); NI: North Indian; SI: South Indian; WI:Western Indian.

Translation of these allelic patterns into predicted phenotypes (Figure 2; Supplementary Table S4) reveals clinically relevant distributions. The 53.8% normal metabolizer frequency falls within global ranges (42%–67%) but is lower than that of some Indian subpopulations, such as Gujaratis (84.7%) (Kane, 2021; Gene-specific Information Tables for CYP2D6; Sivadas et al., 2024; Paradkar et al., 2018; Koopmans et al., 2021). Conversely, the 38.7% prevalence of intermediate metabolizers exceeds most global estimates and is particularly relevant for drugs requiring dose adjustments in IMs. The poor metabolizer frequency (2.2%) aligns with South Asian estimates (∼2.9%) (Kane, 2021; Gene-specific Information Tables for CYP2D6; Gaedigk et al., 2017; Koopmans et al., 2021; Dhuya et al., 2020) but shows state-wise variability in India, ranging from 1.8% in Andhra Pradesh to 4.8% in Kerala (Dhuya et al., 2020; Lamba et al., 1998; Abraham et al., 2000a; Abraham et al., 2000b; Gogtay et al., 2014). The prevalence of ultrarapid metabolizers (5.3%) further underscores the importance of CNV-inclusive genotyping, as conventional SNP-only approaches would miss this clinically relevant subgroup.

Taken together, these findings demonstrate that the Central Indian population has a distinct *CYP2D6* genetic profile characterised by a high burden of decreased-function and nonfunctional alleles, moderate gene duplication rates, and a resulting metabolizer phenotype distribution that supports the need for genotype-guided prescribing for nearly half of this population.

### CYP2C19 metabolizer phenotypes distribution and comparison, with other global populations

The *CYP2C19* enzyme plays a critical role in the metabolism of several commonly prescribed antidepressants, particularly SSRIs such as escitalopram, sertraline, and citalopram (Bousman et al., 2023; Hicks et al., 2015). Our findings reveal a *CYP2C19* genetic profile in Central India characterised by a high prevalence of clinically actionable variants, as visually represented in the combined allele frequency heatmap (Figure 1; Supplementary Table S3).

The distribution of key *CYP2C19* alleles shows distinct population patterns. The non-functional CYP2C19*2 allele frequency of 34.6% in our cohort aligns with the broader South Asian range (32%–36%) (Umamaheswaran et al., 2014; Chenchula et al., 2024a) and substantially exceeds the frequencies observed in European (13%), American (17.5%), and African (18.1%) populations (Koopmans et al., 2021; Gene-specific Information Tables for CYP2C19). This high *2 prevalence is clinically relevant given that 62.3% of our study participants were prescribed *CYP2C19*-metabolised antidepressants (sertraline 34.1%, escitalopram 28.2%), where poor or intermediate metabolizer status can directly influence therapeutic response and risk of adverse effects. (Figure 1; Supplementary Table S3).

The increased-function *CYP2C19* *17 allele frequency of 13.7% places Central India within the reported range for Indian populations (13%–19%) (Umamaheswaran et al., 2014; Chenchula et al., 2024a), but lower than the frequencies observed in European (21.6%) and African (42.6%) populations (Koopmans et al., 2021; Gene-specific Information Tables for CYP2C19). This intermediate prevalence underscores the need to consider both loss-of-function and gain-of-function alleles when determining optimal drug dosing. (Figure 1; Supplementary Table S3).

**FIGURE 3:**
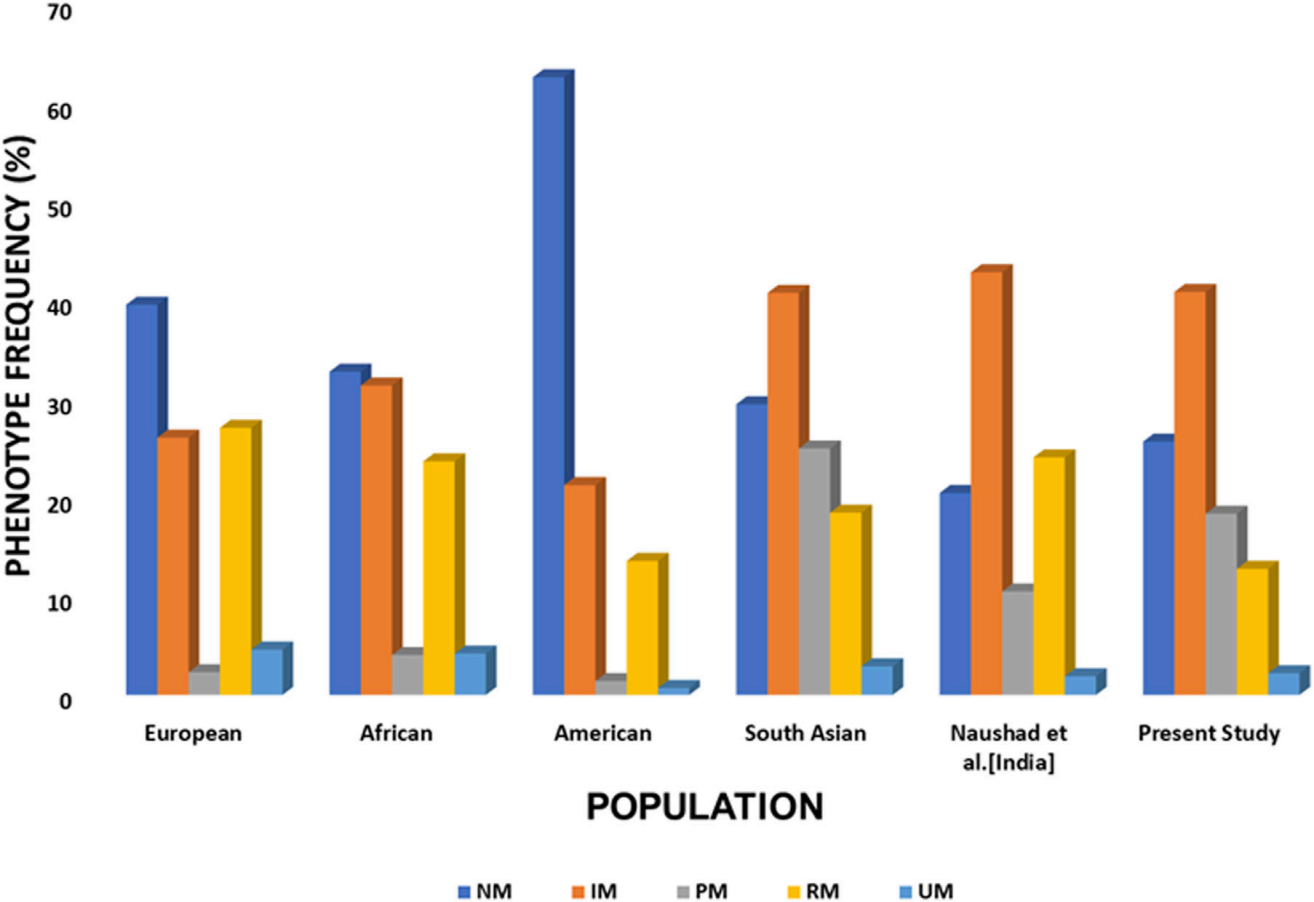
CYP2C19 phenotype distribution across populations. Grouped bar plot showing the proportion of normal metabolizer (NM), intermediate metabolizer (IM), poor metabolizer (PM), rapid metabolizer (RM), and ultrarapid metabolizer (UM) phenotypes for CYP2C19 across the Central Indian cohort, other Indian populations, and global populations. Abbreviations: CI: Central Indian (current study); NI: North Indian; SI: South Indian; WI: Western Indian.

The resulting metabolizer phenotype distribution (Figure 3; Supplementary Table S4) reveals striking clinical implications: only 25.7% of participants were classified as normal metabolizers (NMs), while 74.2% carried non-normal phenotypes (IM, PM, RM, or UM) that would warrant dose adjustment or alternative therapy according to CPIC guidelines. This aligns with the observation by Koopmans et al. that Indian populations carry the highest global likelihood (80.1%) of non-normal *CYP2C19* phenotypes (Koopmans et al., 2021).

The poor metabolizer (PM) prevalence of 18.5% in our cohort is particularly noteworthy, as it significantly exceeds rates in Europeans (2%–3%), Americans (2.3%), and Africans (1.2%) (Koopmans et al., 2021; Gene-specific Information Tables for CYP2C19). This has direct clinical implications for SSRIs like escitalopram, where CPIC guidelines strongly recommend dose reduction or alternative drug selection for PMs. Similarly, the rapid (12.8%) and ultrarapid (2.1%) metabolizer subgroups require consideration of dose increases or alternative therapy to avoid subtherapeutic drug levels.

Our phenotype distribution is consistent with other Indian studies reporting NM frequencies between 20.5% and 27.9% (Naushad et al., 2021; Ghodke et al., 2007; Bhat et al., 2024), which reinforces the stability of CYP2C19 allele distributions across different Indian subpopulations, despite varying cohort characteristics such as healthy volunteers (Naushad et al., 2021), cardiovascular patients (Bhat et al., 2024), and our CMD cohort. Although environmental exposures or epigenetic factors may influence gene expression, there is no evidence that depression or anxiety alters CYP gene frequencies or that CYP genotypes affect CMD susceptibility (Wu et al., 2019; Alshaya, 2022). Comparable findings across multiple Indian studies further strengthen the generalizability of our results (Chenchula et al., 2024a). Collectively, these findings indicate that a substantial proportion of the Central Indian population carries clinically actionable *CYP2C19* phenotypes. When combined with the high prescription rate of *CYP2C19*-substrate antidepressants in this population, these data strongly support the clinical utility of pharmacogenetic testing to optimise antidepressant therapy and reduce the risk of treatment failure and adverse effects (Figure 3; Supplementary Table S4).

### Clinical significance of findings

Genetic variation in *CYP2D6* and *CYP2C19* enzymes can significantly affect drug exposure, efficacy, and safety, ultimately influencing treatment outcomes in psychiatric care, particularly for antidepressants and antipsychotics (Santenna et al., 2024; Zanger and Schwab, 2013; Bousman et al., 2023). More than half of the psychotropic drugs (63%) have prescribing guidelines for dosing linked to CYP2C19 and/or CYP2D6, as developed by these international expert groups (Brown et al., 2025). The implementation of PGx-guided antidepressant therapy has been endorsed by leading international bodies, including the CPIC and the Dutch Pharmacogenomics Working Group (DPWG), both of which provide robust, evidence-based guidelines to aid clinicians in interpreting PGx results and optimising drug therapy (Bousman et al., 2023; Hicks et al., 2015; Brown et al., 2025; Hicks et al., 2017; Beunk et al., 2024). Additional support comes from national PGx networks and databases such as ClinPGx, the Canadian Pharmacogenomics Network for Drug Safety (CPNDS), the French National Network of Pharmacogenetics (RNPGx), and the American College of Medical Genetics and Genomics (ACMG), all of which advocate for personalised prescribing practices based on genotype–phenotype correlations related to these two CYP genes (Whirl-Carrillo et al., 2012; Amstutz et al., 2011; Canadian, 2020; Picard et al., 2017).

Furthermore, major regulatory authorities, such as the European Medicines Agency (EMA) and the U.S Food and Drug Administration (FDA), have also incorporated PGx information, particularly related to *CYP2D6* and *CYP2C19*, into drug labels for numerous medications, reinforcing the clinical importance of PGx-informed prescribing (European Medicines Agency (EMA), 2012; U.S. Food and Drug Administration). Notably, some medications, such as eliglustat and codeine, now require mandatory CYP2D6 genotype-based prescribing according to the FDA recommendations (Kane and Dean, 2012). Among antidepressants, *CYP2D6-*based recommendations are given for paroxetine, venlafaxine, vortioxetine, and *CYP2C19* is implicated in guidelines for citalopram/escitalopram, sertraline, whereas the dosing of most of the TCAs is now linked to variations in either or both of these genes (Bousman et al., 2023; Hicks et al., 2015; Brown et al., 2025; Hicks et al., 2017; Beunk et al., 2024). Several other drugs, including clopidogrel, voriconazole, brivaracetam, pantoprazole for CYP2C19, and brexpiprazole, clozapine, geftinib, and olicerdine, among others, have genotype-informed guidance in their labels for CYP2D6 (U.S. Food and Drug Administration). Actionable recommendations are available based on specific allelic variants and predicted metabolizer phenotypes. Despite global PGx integration, India lacks the implementation infrastructure for this (Naushad et al., 2021). However, the real-world clinical utility of these recommendations is shaped by population-specific allele frequencies. Therefore, understanding the prevalence of actionable PGx variants in diverse ethnic and regional subpopulations, such as this Central Indian population, is essential for identifying individuals most likely to benefit from pharmacogenetic testing. Our study addresses this gap by providing population-specific data showing that almost half (46.2%) of the individuals in our Central Indian cohort would benefit from *CYP2D6*-guided dosing, while three-fourths (74.2%) would require *CYP2C19*-guided dosing.

Depending on ethnicity, 37%–96% of individuals carry at least one clinically actionable *CYP2C19* variant, and 35%–73% carry an actionable *CYP2D6* variant, defined as those for which deviations from standard prescribing are recommended (Gene-specific Information Tables for CYP2D6; Gene-specific Information Tables for CYP2C19; Brown et al., 2025). In our study, overall, 86% of individuals carried at least one actionable PGx variant genotype, providing strong support for the role of PGx testing. These findings align with a whole-genome sequencing-based study by Sahana A et al., which estimated that each Indian individual carries, on average, eight clinically actionable pharmacogenomics (PGx) variants, highlighting the significant PGx variant burden in the Indian population and supporting the broader implementation of PGx-guided prescribing strategies (Sahana et al., 2022). It is also in agreement with global population-based studies that demonstrate the high prevalence of actionable PGx variants. For example, nearly all participants in the UK Biobank (100%) and the U.S. Veterans Health Administration (99%) were found to harbour at least one clinically actionable PGx genotype (Li et al., 2023; Chanfreau-Coffinier et al., 2019). Similarly, a nationwide Swiss study reported a prevalence of 97.3% of actionable variants (Hodel et al., 2024). Supporting this, the ASPREE trial, conducted by Bousman et al., demonstrated that 98.8% of older adults carried at least one actionable PGx genotype, emphasising the clinical potential of PGx-guided prescribing to enable meaningful therapeutic modifications (Bousman et al., 2025). Similarly, a recent study from Japan estimated that approximately one in four patients may benefit from pre-emptive PGx testing when initiating antidepressant therapy, further reinforcing the global relevance of genotype-guided prescribing (Hatano et al., 2025). Collectively, these findings translate large-scale genomic data into practical, region-specific prescribing strategies. They provide strong support for the integration of PGx testing into clinical practice, aiming to reduce trial-and-error approaches, prevent adverse drug reactions, and enable more precise and effective therapy in genetically diverse populations.

### Actionable pharmacogenetic profiles and antidepressant dosing recommendations as per CPIC guidelines

The high prevalence of non-normal metabolizers (46.2% for *CYP2D6* and 74.2% for *CYP2C19*) carries significant implications for antidepressant prescribing in this population. To quantify the potential clinical impact, we cross-referenced the predicted phenotypes with antidepressant prescription data, which revealed a high usage of medications metabolised by these enzymes: sertraline (34.1%), escitalopram (28.2%), and paroxetine (21.2%). For *CYP2D6, we* found that 38.7% of our participants were IMs, for whom CPIC recommends considering a lower starting dose and slower titration for paroxetine (strength of recommendation: optional) and for amitriptyline and nortriptyline, with moderate-strength recommendations suggesting reduced starting and maintenance doses (Hicks et al., 2015; Hicks et al., 2017). *CYP2D6* PMs, accounting for 2.2% of participants, are advised to receive 50% dose reductions for paroxetine (moderate) and fluvoxamine (optional), and to consider alternative drugs for venlafaxine (optional) and for amitriptyline and nortriptyline (strong) (Hicks et al., 2015; Hicks et al., 2017). For the 5.3% UMs, there are strong recommendations to avoid paroxetine and TCAs and to consider alternative medications (Hicks et al., 2015; Hicks et al., 2017).

For *CYP2C19*, the 18.5% PMs are strongly recommended to avoid escitalopram or to consider dosage adjustments with a 50% reduction of the standard maintenance dose, while moderate recommendations apply to dose adjustment with a 50% reduction of the standard maintenance dose for sertraline and amitriptyline (Lamba et al., 1998; Whirl-Carrillo et al., 2012). RMs (12.8%) may benefit from higher doses or alternative agents, such as escitalopram and amitriptyline (optional) (Hicks et al., 2015; Hicks et al., 2017). Collectively, 7.5% of the study population carrying *CYP2D6* variants fall under CPIC’s recommendation to select an alternative drug for paroxetine, venlafaxine, vortioxetine, amitriptyline, and nortriptyline, and 20.6% of patients carried a *CYP2C19* variant with the same recommendation in place of sertraline (UM/PM) and escitalopram (UM/PM), and amitriptyline (UM/PM) (Hicks et al., 2015; Hicks et al., 2017). Table 3 provides a summary of these drug-gene pairings, along with the related CPIC recommendation strengths (Hicks et al., 2015; Hicks et al., 2017).

**TABLE 3:** Drug-gene pairs with corresponding CPIC guidelines-based recommendations for antidepressants for the study population.

CPIC recommendations	Proportion of patients with CYP2D6 PGx recommendations (%)	Proportion of patients with CYP2C19 PGx recommendations (%)
Lower starting dose and slower titration schedule, and lower maintenance dose than normal metabolizers (IM)	38.7% (Paroxetine: Optional)	
Initiate therapy with the recommended starting dose. Slower titration schedule and lower maintenance dose than normal metabolizers (IM)		40.9% (Escitalopram: Moderate Sertraline: Moderate)
25% reduction of the recommended starting dose and utilise therapeutic drug monitoring to guide dose adjustments (PM)	38.7% (Amitriptyline: Moderate Nortriptyline: Moderate)	
50% reduction in recommended starting dose, slower titration schedule, and a 50% lower maintenance dose (PM)	2.2% (Paroxetine: Moderate Vortioxetine: Moderate Fluvoxamine: Optional)	18.4% (Escitalopram: Strong Sertraline: Moderate Amitriptyline: Moderate)
Select an alternative drug (UM/PM)	7.5% (Paroxetine: Moderate Venlafaxine: Optional Vortioxetine: Optional Amitriptyline: Strong Nortriptyline: Strong)	20.6% (Escitalopram: Strong Sertraline: Moderate Amitriptyline: Optional)
Start with the recommended dose; if response is inadequate with maintenance dose, increase to a higher maintenance dose or switch to an appropriate alternative antidepressant (RM)	-	12.8% (Escitalopram-Optional Amitriptyline: Optional)

Our findings move beyond theoretical allele frequencies to demonstrate a tangible and pressing clinical challenge. The convergence of a high prevalence of actionable pharmacogenetic variants with the common prescription of corresponding medications creates a substantial risk for drug-gene interactions. Some alleles in both Genes have deviated from the HWE, which provides robust statistical evidence for significant population substructure within our Central Indian cohort, reflecting the known genetic heterogeneity of the region, which encompasses numerous distinct groups, including Scheduled Tribes (e.g., Gond, Bhil) with varying proportions of Indo-European, Dravidian, and Austroasiatic ancestry (Mastana, 2014; Sharma et al., 2012; De Meeûs, 2018). India accounts for nearly 18% of the global population and carries a substantial share of the global mental health burden. Our study provides the essential first step in quantifying the prevalence of actionable pharmacogenetic variants within a real-world clinical population and aligning it with contemporary prescribing data. This effectively maps the ‘genetic landscape’ and identifies the population at risk, thereby establishing a strong rationale and a defined cohort for future longitudinal studies aimed at directly demonstrating the clinical utility and cost-effectiveness of either pre-emptive or reactive genotyping testing in this setting to enhance treatment outcomes and reduce adverse drug reactions (Chenchula et al., 2024b).

### Practical considerations for implementing PGx testing in the indian healthcare context

Pharmacogenetics holds significant promise for improving therapeutic outcomes and optimising healthcare resource utilisation in low- and middle-income countries (LMICs) such as India. However, several barriers hinder its widespread implementation. These include limited population-specific data on the prevalence of clinically actionable variants, lack of large-scale studies correlating genotypes with drug response and adverse events, scarcity of cost-effectiveness analyses, and infrastructural as well as technological constraints. Additional challenges include the absence of India-specific pharmacogenomics (PGx) guidelines, policies, and regulatory frameworks, as well as limited awareness and training among clinicians.

The cost of comprehensive pharmacogenetic testing, which covers most CPIC Level A drug-gene pairs, is approximately USD 70–80. This may not be affordable for the majority of patients, given the predominantly out-of-pocket health expenditure in India. Furthermore, most PGx testing facilities are operated by private laboratories, with limited availability in government institutions where subsidised testing could enhance access. Geographical inequity is another concern, as most testing facilities are concentrated in metropolitan and tier-1 cities, with very limited availability in district hospitals and rural settings.

Despite these challenges, several opportunities can be leveraged. India’s growing genomic datasets (such as IndiGenomes) provide a valuable foundation for population-specific allele frequency estimation. Public–private partnerships and targeted testing strategies for high-impact medications (e.g., anticancer, antiplatelet, antidepressant drugs) could improve affordability and clinical adoption. A phased implementation beginning in government tertiary-care centres, supported by digital platforms for reporting and clinical decision support, could enable smooth integration into routine practice. Our centre is the only tertiary care Government Institute which has initiated a pilot implementation program offering PGx testing at a subsidised cost, which has demonstrated feasibility and improved patient access. In the long term, broader adoption of PGx testing could yield substantial cost savings by reducing trial-and-error prescribing, minimising ADRs, and improving overall healthcare outcomes, making it a strategic investment for the Indian healthcare system.

### Study limitations

While our study provides important insights into CYP variation in Central India, several limitations should be taken into consideration. First, our analysis was limited to nine clinically relevant Tier one alleles of CYP2D6 and CYP2C19. The selection of alleles was informed by their known biogeographical distribution to maximise relevance for the South Asian population. The excluded Tier one alleles, such as CYP2D6 ∗9, ∗17, and ∗29, are predominantly prevalent in African and other populations, with existing data suggesting they are exceedingly rare or absent in South Asian groups (Baldacci et al., 2023). The frequency of CYP2D6*9 in previous Indian studies is also very low (0.2%), and *17 and *29 was absent (Umamaheswaran et al., 2014; Jain et al., 2021). Therefore, their exclusion was a targeted decision, and the resulting underestimation of actionable genotypes is expected to be minimal. While these alleles capture the most common and clinically impactful variants, we acknowledge that both genes possess extensive genetic diversity, including rare alleles and complex structural variants not interrogated by our SNP-based assay, such as the *36+*10 hybrid, *13, and *14. This targeted approach may lead to the misclassification of diplotypes, thereby impacting the accuracy of phenotype predictions. Future work using long-read sequencing or comprehensive genotyping panels would provide more complete haplotype resolution. Second, as a hospital-based sample from a single tertiary care centre, our study population consisted exclusively of individuals diagnosed with common mental disorders from across the central India districts, which may introduce a degree of selection bias, and may not fully represent the entire Central Indian population; however, given that PGx allele frequencies are generally considered stable across disease states and no evidence points to involvement of *CYP2D6* and *CYP2C19* as disease susceptibility genes, these findings are still likely to reflect broader trends within the Central Indian population. However, future true population-based sampling would be ideal for broader generalisations. Third, deviations from the HWE observed for some alleles likely reflect population substructure, rather than technical artefacts, as genotyping reliability was confirmed through positive controls in SNP Genotyping and multiple replicate testing with necessary technical QC measures followed in CNVs; this deviation in HWE pattern is a classic signature of the Wahlund effect, the known genetic heterogeneity of the region, which encompasses numerous distinct groups, including various tribes (Mastana, 2014; Sharma et al., 2012; De Meeûs, 2018). Fourth, *CYP2D6* genotyping in the present study was conducted using two validated genotyping platforms: KASP technology for SNP detection and TaqMan for CNV assessment. However, the final diplotype and metabolizer phenotype assignment were performed using standard CPIC-recommended genotype-to-phenotype translation algorithms, which integrated data from both platforms to ensure accuracy and clinical relevance. Furthermore, we did not perform population ancestry correction (PCA) using genome-wide data, which is the standard approach in population genetics for establishing representativeness. Future studies employing genome-wide SNP data in population-based cohorts will be required to comprehensively delineate population structure and confirm representativeness. Finally, this cross-sectional study was designed to establish the baseline prevalence of actionable pharmacogenetic variants in an underrepresented Central Indian population and did not assess drug–gene associations, treatment outcomes, or ADRs. While this limits direct clinical interpretation, the findings provide a foundational dataset of allele-phenotype associations that can guide future longitudinal studies aimed at linking pharmacogenetic variation to therapeutic response and safety.

## Conclusion

In conclusion, this study represents the first large-scale evaluation of clinically actionable CYP2D6 and CYP2C19 variants in a Central Indian population with common mental disorders. We found that 46.2% of individuals were CYP2D6 non-normal metabolizers, 74.2% were CYP2C19 non-normal metabolizers, and 86% carried at least one actionable pharmacogenetic genotype. These findings highlight a substantial opportunity for implementing genotype-guided prescribing to minimise treatment failures, reduce adverse drug reactions, and optimise dosing strategies.

Importantly, our results demonstrate that pharmacogenomic variability in Central India is both clinically significant and comparable to global patterns, underscoring the need for pre-emptive PGx testing not only for antidepressants but also for other *CYP2D6* and *CYP2C19* metabolised medications. Integrating pharmacogenomic testing into routine psychiatric care, beginning with tertiary care centres and expanding to broader healthcare networks, could markedly improve safety, efficacy, and personalisation of treatment across India’s genetically diverse populations.

## Data Availability

The original contributions presented in the study are included in the article/Supplementary Material, further inquiries can be directed to the corresponding authors.

## Supplementary material

SUPPLEMNTARY FIGURE S1 Geographic Distribution of Native Districts of the Study Population in Central India. Map showing the native districts of participants across Madhya Pradesh and Chhattisgarh. Each dot represents one or more participants, illustrating the broad geographical coverage and regional representation of the cohort.

SUPPLEMNTARY FIGURE S2 Principal Component Analysis (PCA) of CYP2D6 and CYP2C19 Allele Frequencies. PCA plot based on allele frequency data from the Central Indian (CI) cohort and other populations. PC1 and PC2 represent the first two principal components explaining the majority of genetic variation. Clustering shows the genetic relationship of the CI cohort within the broader South Asian context. Abbreviations: CI: Central Indian (current study); NI: North Indian; SI: South Indian; WI: Western Indian.
