# Supplementary figures and images for: Pharmacogenetic variations and clinical implications of actionable CYP2D6/CYP2C19 variants in Central Indian patients with common mental disorders
Source: Front Pharmacol. 2025 Nov 25;16:1697866. doi: 10.3389/fphar.2025.1697866 (PMC12685848; doi:10.3389/fphar.2025.1697866)

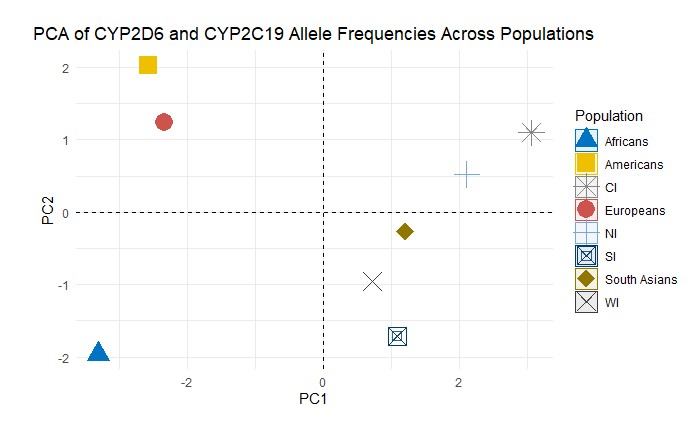

Supplement: Supplementary file 1 [file Image2.tif]

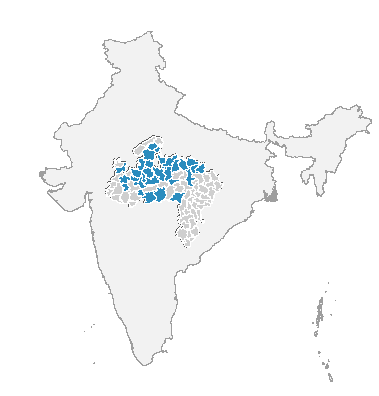

Supplement: Supplementary file 2 [file Image1.tif]
