# Supplementary material for: Pharmacogenetic variations and clinical implications of actionable CYP2D6/CYP2C19 variants in Central Indian patients with common mental disorders
Source: Front Pharmacol. 2025 Nov 25;16:1697866. doi: 10.3389/fphar.2025.1697866 (PMC12685848; doi:10.3389/fphar.2025.1697866)
Supplement: Supplementary file 3 [file DataSheet1.pdf]

**Table S1. CYP2D6 Allele Frequencies Across Populations**

| Allele | Europeans | Africans | Americans | South Asians | Indi Genome [10] | Sivadas A et al. [38] | SI [9] | NI [9] | WI [39] | CI (Present Study) |
|---|---|---|---|---|---|---|---|---|---|---|
| CYP2D6*2 | 34.3 | 26.7 | 32.7 | 36.2 | 37.4 | 21.4 | 32 | 34.8 | 30.8 | 40.9 |
| CYP2D6*2A | 54 | 67 | 47 | 52 | 56.4 | NA | NA | NA | NA | 46.6 |
| CYP2D6*3 | 4.1 | <1 | <1 | <1 | NA | NA | 0 | 9.2 | 0 | 5.7 |
| CYP2D6*4 | 15.5 | 11.9 | 15.7 | 11.6 | 10.9 | 7.8 | 7.3 | 11.5 | 11.5 | 10.4 |
| CYP2D6*6 | 2.2 | <1 | <1 | <1 | NA | NA | NA | NA | NA | 1.9 |
| CYP2D6*10 | <2 | 4–6 | <2 | 21 | 19.2 | 5.2 | 10.2 | 27.2 | 19.2 | 21.6 |
| CYP2D6*41 | 9.0 | 4–11.5 | 4.4 | 15 | 13.2 | 12.6 | 12.3 | 12.5 | 17.3 | 17.3 |

**SI: South Indian; NI: North Indian; WI: West Indian; CI: Central Indian**

**Table S2. Frequency of Copy Number Variations (CNVs) in the CYP2D6 Gene across Populations**

| Allele | Europeans | Africans | Americans | South Asians | Indi Genome [10] | Sivadas A et al. [38] | SI [9] | NI [9] | WI [39] | CI (Present Study) |
|---|---|---|---|---|---|---|---|---|---|---|
| CYP2D6*5 (CNV) | 3–5 | 5.9 | 3–5 | 2.6 | NA | 3.5 | 1.9 | 1.8 | 3.9 | 4.2 |
| *1/*2X≥N (CNV) | 3.6 | 7–16 | 1.5 | 2.5 | NA | 1.4 | NA | 2.5 | 15.4 | 4.1 |

**SI: South Indian; NI: North Indian; WI: West Indian; CI: Central Indian.**

**Table S3. CYP2C19 Allele Frequencies Across Populations**

| Allele | Europeans | Africans | Americans | South Asians | Indi Genome [10] | Naushad et al. [49] | SI [9] | NI [9] | WI [52] | CI (Present Study) |
|---|---|---|---|---|---|---|---|---|---|---|
| CYP2C19*2 | 18.3 | 18.1 | 10.5 | 35.7 | 36.2 | 32 | 32.3 | 35.7 | 41.7 | 37.3 |
| CYP2C1 9*3 | < 1 | < 1 | < 1 | 1.6 | < 1 | < 1 | 1.1 | 1.9 | 1.2 | 2.3 |
| CYP2C19*17 | 22.4 | 23.5 | 12.0 | 17.3 | 14.3 | 13.9 | 19 | 13.3 | 13.7 | 16.1 |

**SI: South Indian; NI: North Indian; WI: West Indians; CI: Central Indian**

**Table S4. CYP2D6 And CYP2C19 Predicted Phenotype Distribution Across the Indian and Global Population**

| **CYP2D6 Phenotypes** | **European (%)** | **African (%)** | **American (%)** | **South Asian (%)** | **Sivadas A et al.(India) (%)** | **Present Study Cohort (%)** |
|---|---|---|---|---|---|---|
| Normal Metabolizer (NM) | 58.9 | 84.03 | 52.8 | 58.2 | 58 | 53.8 |
| Intermediate Metabolizer (IM) | 34 | 24.2 | 13.2 | 28.6 | 28 | 38.7 |
| Poor Metabolizer (PM) | 1.8 | 2.7 | 1.9 | 3.05 | 2.3 | 2.2 |
| Ultrarapid Metabolizer (UM) | 3.3 | 3.8 | 4.6 | 5.6 | 2.0 | 5.3 |
| **CYP2C19 Phenotypes** | **European (%)** | **African (%)** | **American (%)** | **South Asian (%)** | **Naushad et al.(India) (%)** | **Present Study Cohort (%)** |
| Normal Metabolizer (NM) | 39.6 | 32.8 | 62.7 | 29.5 | 20.5 | 25.7 |
| Intermediate Metabolizer (IM) | 26.1 | 31.4 | 21.3 | 40.8 | 42.9 | 40.9 |
| Poor Metabolizer (PM) | 2.3 | 4.05 | 1.4 | 12-25 | 10.5 | 18.4 |
| Rapid Metabolizer (RM) | 27.1 | 23.7 | 13.6 | 18.5 | 24.1 | 12.8 |
| Ultrarapid Metabolizer (UM) | 4.6 | 4.2 | 0.7 | 2.9 | 1.9 | 2.2 |
